# Effect of haemolysis and repeated freeze-thawing cycles on wild boar serum antibody testing by ELISA

**DOI:** 10.1186/1756-0500-4-498

**Published:** 2011-11-16

**Authors:** Mariana Boadella, Christian Gortázar

**Affiliations:** 1 IREC (CSIC-UCLM-JCCM), Ronda de Toledo s/n, 13071 Ciudad Real, Spain

**Keywords:** Aujeszky's disease, Blood sample mishandling, Serological surveillance, Wildlife disease monitoring

## Abstract

**Background:**

Monitoring wildlife diseases is needed to identify changes in disease occurrence. Wildlife blood samples are valuable for this purpose but are often gathered haemolysed. To maximise information, sera often go through repeated analysis and freeze-thaw cycles. Herein, we used samples of clean and haemolysed Eurasian wild boar (*Sus scrofa*) serum stored at -20°C and thawed up to five times to study the effects of both treatments on the outcome of a commercial ELISA test for the detection of antibodies against Suid Herpesvirus 1 (ADV).

**Results:**

The estimated prevalence of antibodies against ADV was 50-53% for clean and haemolysed sera. Hence, haemolysis did not reduce the mean observed serum antibody prevalence. However, 10 samples changed their classification after repeated freeze-thawing. This included 3 (15%) of the clean sera and 7 (41%) of the haemolysed sera.

**Conclusions:**

We recommend (1) establishing more restrictive cut-off values when testing wildlife sera, (2) recording serum quality prior to sample banking, (3) recording the number of freezing-thawing cycles and (4) store sera in various aliquots to reduce repeated usage. For instance, sera with more than 3 freeze-thaw cycles and a haemolysis of over 3 on a scale of 4 should better be discarded for serum antibody monitoring. Even clean (almost not haemolysed) sera should not go through more than 5 freeze-thaw cycles.

## Background

Monitoring wildlife diseases is needed to identify changes in disease occurrence and to measure the impact of intervention. However, obtaining samples from wild animals is difficult as compared to pets or livestock, due to the limited accessibility of the former [1,2]. Wildlife blood samples are often gathered post-mortem from shot (hunter-harvested) animals, and then centrifuged to obtain serum. Occasionally, whole blood samples are obtained from gamekeepers and sent frozen to the laboratory. Sera are stored frozen and often re-used several times in order to maximise the information obtained. In consequence, wildlife sera are often haemolysed and/or go through repeated freeze-thaw cycles (e.g. [3]). However, both haemolysis and freeze-thawing may affect the performance of tests based on serum antibody detection, such as the popular enzyme-linked immunosorbent assay (ELISA).

Recently, a study on the effect of swine blood sample handling on *Erysipelothrix rhusiopathiae *antibody detection by indirect ELISA revealed that serum immunoglobulin G antibodies were stable in the face of several sample mishandling events, including repeated freeze-thawing and minimal to severe haemolysis. Only samples simulating extreme haemolysis (100% haemolysed whole blood) had significantly lower optical density (OD) readings. However, haemolysis and freeze-thawing were not studied in combination, and the effect of such treatments on antibodies against other disease agents is unknown [4].

Herein, we used samples of clean and haemolysed Eurasian wild boar (*Sus scrofa*) serum stored at -20°C and thawed up to five times to study the effects of both treatments on the outcome of an ELISA test for the detection of antibodies against Suid Herpesvirus 1 (ADV), the aetiological agent of Aujeszky's disease. Based on the abovementioned results for *E. rhusiopathiae *in pigs, we expected no strong effect of haemolysis and freeze-thawing on test performance.

## Results

A total of 20 sera were analyzed clean and from these, 17 could also be tested haemolysed (Table 1). The ELISA results coincided in 14 cases (8 positive, 6 negative; 82%). Two negative clean sera tested positive and doubtful, respectively, with haemolysis, and one positive clean sera tested negative with haemolysis. The estimated prevalence of antibodies against ADV was 10 of 20 (50%; 29-70 95% CI) and 9 of 17 (53%; 29-75 95% CI) for clean and haemolysed sera, respectively. Hence, haemolysis did not reduce the observed serum antibody prevalence (χ^2 ^= 0.032, 1df, p > 0.05).

**Table 1:** Classification of wild boar sera through five freeze-thaw cycles.

	Clean					Haemolysed				
Freeze/thaw cycle	**1**	**2**	**3**	**4**	**5**	**1**	**2**	**3**	**4**	**5**

Sample number										
**1**	1	1	1	1	1	1	1	1	1	1
**2**	1	1	1	1	1	1	1	1	1	1
**3**	0	0	0	0	0	0	0	0	0	2
**4**	0	0	0	0	2	0	0	0	0	2
**5**	0	0	0	0	0	0	0	0	0	0
**6**	1	1	1	1	1	1	1	1	1	1
**7**	1	1	1	1	1	1	1	1	1	1
**8**	0	0	0	0	0	0	0	2	0	2
**9**	1	1	1	1	1	0	0	0	2	1
**10**	1	1	1	1	1	1	1	1	1	1
**11**	0	0	0	0	0	1	1	1	1	1
**12**	1	1	1	1	1	1	1	1	1	1
**13**	0	2	0	0	0	2	2	0	0	0
**14**	1	1	1	1	1	1	1	1	1	1
**15**	0	0	0	0	0	0	0	0	0	2
**16**	1	1	1	1	1	1	1	1	1	1
**17**	0	0	0	0	0	0	0	0	2	2
**18**	0	0	0	0	2					
**19**	0	0	0	0	0					
**20**	1	1	1	1	1					

**N° positive samples**	10	10	10	10	10	9	9	9	9	10
**N° negative samples**	10	9	10	10	8	7	7	7	6	2
**N° doubtful samples**	0	1	0	0	2	1	1	1	2	5
**N° changing state**		1	0	0	2		0	2	3	7
**% changing state**		5%	0%	0%	10%		0%	12%	18%	41%

Classification of the 20 wild boar sera samples (20 clean; 17 haemolysed) after 1 to 5 freeze-thaw cycles into positive (1), negative (0) or doubtful (2) according to a commercial ELISA for the detection of serum immunoglobulin G antibodies against Aujeszky's disease virus.

**Figure 1:**
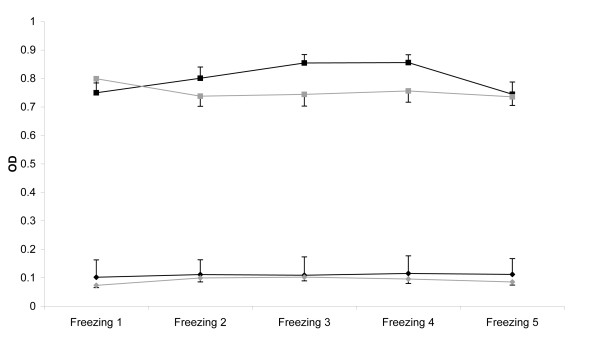
**Optical densities (OD) through five freeze-thaw cycles**. Mean optical densities (OD) for positive (black diamonds) and negative (black squares) clean sera, and for positive (grey diamonds) and negative (grey squares) haemolysed sera (± standard error, SE) through the five freeze-thaw cycles (Freezing 1 to 5).

Table 1 shows the outcome of the experimental manipulation of 37 wild boar sera in terms of ELISA test results. Only 3 (15%) of the clean sera changed their result after repeated freeze-thawing, changing from negative to doubtful (1 case at the 2^nd ^cycle and 2 cases at the 5^th^). In contrast, 7 (41%) of the haemolysed sera changed their result (2 cases at the 3^rd ^cycle, 3 at the 4^th ^and 7 at the 5^th^). These changes occurred between negative and doubtful, except for one case. This one consisted of a serum testing first three times negative, then doubtful and finally positive. All 10 sera with changes at any cycle (27% of 37) tested negative (9) or doubtful (1) in the first time, while all 19 sera that tested positive at the first cycle (10 clean sera and 9 haemolysed sera) maintained their positivity through the five freeze-thaw cycles. Figure 1 shows the mean optical density readings for clean and haemolysed sera during the five freeze-thaw cycles.

## Discussion

Based on the results obtained in this experiment, we rejected our initial hypothesis: Haemolysis alone and the combination of haemolysis and freeze thawing affected the results of the ADV ELISA test. These observations have implications for wildlife disease monitoring based on serum antibody detection.

The effect of the described sample mishandling events was mostly a higher rate of doubtful results. Based on our observations, we recommend (1) establishing more restrictive cut-off values when testing wildlife sera, (2) recording serum quality prior to sample banking (3) recording the number of freezing-thawing cycles and (4) store sera in various aliquots to reduce repeated usage. Regarding the cut-off, we observed a trend towards lower ODs with increasing number of freeze-thawing cycles. So, if the cut off was maintained as the one defining a positive sample (i.e. considering any doubtful samples as negative), the result in terms of prevalence (number of positive samples divided by total number of tested samples) would not change except for one single case at one single cycle. Regarding the possibility to classify sera based on their apparent quality, a subjective haemolysis-scale from 1 (almost nil) to 4 (severe), could be defined. Additionally, the number of freeze-thaw cycles can be recorded for each sample aliquot. For instance, sera with more than 3 freeze-thaw cycles and a haemolysis of over 3 on a scale of 4 should better be discarded for serum antibody monitoring. Even clean (almost not haemolysed) sera should not go through more than 5 freeze-thaw cycles.

In studies on domestic animals, samples are normally ELISA tested in duplicate to reduce within-laboratory variability (e.g. [5]). This is not always the case in wildlife, particularly if the available serum volume is limited and several different tests are run (e.g. [6,7]). Running each serum in duplicate also means duplicating the cost per individual test, particularly when using commercial ELISA kits. However, efforts should be done to test all wildlife sera in duplicate in order to reduce variability (e.g. [8]).

As in Neumann and Bonistalli's [4] study on *E. rhusiopathiae *antibody detection in pigs, our results on ADV antibody detection in wild boar are not directly applicable to other host-pathogen binomia. It is however advisable to take care in the interpretation of ELISA results obtained from poor quality samples. Efforts should be made to evaluate the specific effects of thawing and haemolysis on the results of other antibody detection tests.

## Methods

### Samples

Blood samples were collected from 20 stalking-harvested wild boar. Blood was drawn from the thoracic major veins by exsanguination immediately after death. Two blood collection tubes were filled per animal and stored at 4°C during transport until the laboratory; although for three animals only one tube could be filled. Serum was obtained by centrifugation from one of the tubes and stored at 4°C. The second tube was used to obtain haemolysed serum after freezing the whole blood simulating the treatment that occurs when blood samples are sent frozen. In a subjective haemolysis-scale from 1 (almost nil) to 4 (severe), the haemolysed obtained sera were classified as level 3. After obtaining all fresh and haemolysed sera, the first ELISA test was performed. Sera were stored at -20°C for one week and thawed at 4°C for one night. One hour before performing the ELISA analysis, sera were removed from 4°C storage and brought to room temperature. After analysis, sera were frozen again to -20°C. This process was repeated 5 times.

All animal samples used for this study came from opportunistic sampling during legal hunting. No life animal was handled and no special permits were required.

### ELISA test

A commercially available blocking ELISA was used for detection of antibodies to the gpI antigen of Suid Herpesvirus 1 (IDEXX HerdCheck Anti-ADV gpI, IDEXX, Inc., USA). This ELISA technique has been broadly used for testing antibodies to ADV in different wild boar populations [9-11].

The ELISA was performed following the manufacturer's instructions, in an ADV antigen-coated microwell plate, using a 1:2 serum dilution. One hundred microliters of diluted sera were added in each microwell and incubated for 1 hour at room temperature (RT). Samples, positive and negative controls were tested in duplicate in each plate. Subsequent to a wash step, 100 μl of anti-ADVgpI monoclonal antibody conjugate was added and incubated at RT for 20 minutes. If no gpI antibodies were present in the tested serum, the conjugated gpI antibodies were free to react with the gpI antigen. Conversely, if gpI antibodies were present in the tested serum, the enzyme-conjugated monoclonal antibodies were blocked from reacting with the antigen. Following the incubation, the unreacted conjugate was washed out and the reaction was revealed by adding 100 μl of substrate/chromogen solution. In the presence of substrate enzyme, reaction generated blue colour. After 15 minutes of revealing, the reaction was stopped with 50 μl/well of Stop solution and optical density (OD) was measured in a spectrophotometer at 650 nm.

Results were expressed as a percentage of inhibition (%IN) value using the following formula: [%IN = (mean negative control OD - mean sample OD/mean negative control OD) × 100]. The quantity of antibodies to ADV-gpI was inversely proportional to the OD and directly proportional to the %IN. According to the manufacturer's instructions, only samples with %IN values equal to or greater than 40% were considered positive. Samples with a %IN value between 30-40% were considered doubtful and sera with %IN values < 30% were classified as negative.

## List of abbreviations

ADV: Aujeszky disease Virus, Suid Herpervirus I; ELISA: enzyme-linked immunosorbent assay; OD: Optical density; RT: room temperature.

## Competing interests

The authors declare that they have no competing interests.

## Authors' contributions

MB collected the samples of the study and carried out the laboratory work. MB and CG analyzed the data and drafted the manuscript. Both authors read and approved the final manuscript.
